# Endophenotypes of Late‐Onset Alzheimer's Disease in Mouse Models Expressing IL1RAP Risk Alleles

**DOI:** 10.1002/alz70855_102802

**Published:** 2025-12-23

**Authors:** Kevin P Kotredes, Ravi S Pandey, Michael Sasner, Adrian L Oblak, Stacey J Sukoff Rizzo, Paul R Territo, Gregory W Carter, Bruce T. Lamb, Gareth R Howell, MODEL‐AD

**Affiliations:** ^1^ The Jackson Laboratory, Bar Harbor, ME, USA; ^2^ Stark Neurosciences Research Institute, Indiana University School of Medicine, Indianapolis, IN, USA; ^3^ University of Pittsburgh School of Medicine, Pittsburgh, PA, USA; ^4^ The Jackson Laboratory for Genomic Medicine, Farmington, CT, USA

## Abstract

**Background:**

It is estimated that by 2050 the number of Alzheimer's disease (AD) patients will exceed 12 million, 95% of which are sporadic, late‐onset AD (LOAD). Without novel therapeutics, unlike other age‐related disorders, LOAD mortality will continue to increase. But as next‐generation‐sequencing technologies improve, human disease risk factors are emerging, correlated to the prevalence and severity of disease. Alterations in the *IL1RAP* gene are found to be a strong risk factor for amyloid accumulation in LOAD patients. IL1RAP is a membrane‐bound IL‐1ß receptor expressed in most tissues, including CNS‐specific astrocytes, microglia, and neurons, relaying immune signals via NF‐κB and MAPK transcription factors. Further, a neuron‐specific isoform, *IL1RAPb*, has been identified and found to disrupt MYD88 scaffolding. As a suspected regulatory mechanism for IL‐1ß vulnerability, IL1RAP may prove to be a potential target for intervention against AD.

**Methods:**

Mouse models of LOAD are underutilized and in short supply. Most popular AD strains express amyloid‐related, familial AD transgenes and fail to address the heterogeneous disregulation observed in human disease. For better preclinical models of LOAD, MODEL‐AD has developed novel mouse strains incorporating genetic and environmental risk factors identified from human data sets. To investigate how IL1RAP specifically impacts disease, we have designed two strains expressing loss‐of function *Il1rap* transcripts‐ a whole body *Il1rap* knockout and a neuron‐specific *Il1rapb* conditional knockout.

**Results:**

We generated mouse strains on a LOAD‐relevant genetic background (APOEε4, Trem2*R47H, and humanized *App*) and knocked‐out *Il1rap* or *I1rapb* transcripts which produced gene expression signatures in the brain more similar to those seen in human AD patients. To exacerbate this phenotype, we utilized sterile‐infection and high‐fat diet insults which produced normal immune responses evidenced by increases in plasma TNFα, IL‐6, and IL1RAP agonist, IL‐1ß. Both strains also showed an increase in peripheral biomarker neurofilament light chain (NfL), suggesting downstream neuronal injury. More detailed, CNS‐specific characterization of the many aspects of IL1RAP function are still in progress.

**Conclusion:**

Here we present two novel mouse strains of LOAD, expressing human‐relevant genetic backgrounds aimed at investigating IL1RAP function as a model of human disease and potential target for therapeutic intervention against neurodegeneration.

